# Functional divergence and stage-specific symbiosis of endophytic *Tulasnella* fungi in the endangered orchid *Paphiopedilum malipoense*

**DOI:** 10.1080/15592324.2026.2689804

**Published:** 2026-06-19

**Authors:** Shan Du, Xiao-feng Liao, Fan Tian, Lang Huang, Tang-hang Wei, Da Huo, Tao Xie

**Affiliations:** a Guizhou University, Guiyang, Guizhou, China; b Guizhou Academy of Forestry/Key Laboratory of National Forestry and Grassland Administration on Biodiversity Conservation in Karst Mountainous Areas of Southwestern China, Guiyang, Guizhou, China; c Guizhou Provincial Botanical Garden, Guiyang, Guizhou, China; d Wangmo Forestry Bureau, Wangmo, Guizhou, China

**Keywords:** *Paphiopedilum malipoense*, *Tulasnella*, mycorrhizal specificity, symbiotic germination, stage-specific symbiosis, functional complementarity

## Abstract

*Paphiopedilum malipoense*, a critically endangered orchid, depends entirely on mycorrhizal fungi for germination, complicating its conservation. This study isolated 12 fungal strains from wild roots, all identified as *Tulasnella*, confirming strict host specificity. Cross-species germination assays revealed functional divergence: strains MLP116, MLP027, and MLP232 supported full protocorm-to-seedling development with germination rates of 54.27%, 54.88%, and 62.01%, respectively, while others induced developmental arrest. Seedling symbiosis showed stage-specific effects: MLP217, ineffective during germination, increased seedling biomass by 131%. MLP232 performed excellently in both stages, achieving the highest germination rate and 98% biomass increase, with elevated IAA and soluble protein. Physiological profiling demonstrated functional complementarity: MLP161 enhanced nutrient acquisition; MLP246 boosted chlorophyll and antioxidants; and MLP281 increased antioxidant activity but suppressed growth. These results indicate that part *Tulasnella* strains exhibit stage-specific efficiency and functional complementarity. We recommend using tailored fungal consortia in orchid conservation to synergistically support complete life cycle development, providing a practical framework for safeguarding endangered species like *P. malipoense*.

## Introduction

Mycorrhizal fungi represent a core microbial group that forms long-term, stable, and mutualistic relationships with plants. In terrestrial ecosystems, approximately 85% of vascular plants engage in nutrient exchange and ecological adaptation through the formation of mycorrhizal structures.^1^ Mycorrhizal fungi play a pivotal role in this process: they not only significantly enhance plant uptake of mineral nutrients and water and improve stress resistance, but also broadly participate in ecosystem carbon, nitrogen, and phosphorus cycling.^2-5^ Based on morphology, colonization patterns, and function, mycorrhizae can be classified into two main categories: ectomycorrhizae and endomycorrhizae. Endomycorrhizae further encompass specialized types such as arbuscular mycorrhizae, ericoid mycorrhizae, and orchid mycorrhizae.^1^ Notably, orchids have evolved a characteristic mycoheterotrophic life history, where seed germination and seedling establishment strictly depend on specific mycorrhizal fungal partners, highlighting the central role of these endomycorrhizal fungi in their life cycle.^6^ ^,^ ^7^ During the initial protocorm stage, the plant lacks photosynthetic capacity and must acquire all necessary nutrients through mycorrhizal fungi.^8^ As development progresses, this nutritional dependency may persist into adulthood, albeit with varying degrees among species.^9^ ^,^ ^10^ Known orchid mycorrhizal fungi primarily belong to taxa such as *Tulasnella*, *Ceratobasidium*, and *Serendipita,* ^11-13^ demonstrating notable host specificity—a characteristic particularly pronounced in *Paphiopedilum* species and most stringent during germination.^14-16^ Importantly, symbiotic effectiveness is strain-dependent; only specific fungal strains promote germination and development,^17^ ^,^ ^18^ while incompatible isolates may induce growth arrest.^16^ ^,^ ^19^ This strict specificity poses a significant bottleneck for the conservation of endangered orchids, as the identification of functionally effective fungal partners is paramount.

*Paphiopedilum malipoense*, an endemic species of karst limestone regions, occupies a phylogenetically pivotal position within *Paphiopedilum* and serves as valuable material for studying orchid evolution.^20^ This species is of significant horticultural interest due to its distinctive leaf mottling, chartreuse to greenish-yellow flowers, and extended flowering period.^21^ However, habitat degradation and over-collection have driven it to the brink of extinction,^22^ leading to its classification as a National first-class protected plant in China and its inclusion in CITES Appendix I.^23^ The species' seed endosperm deficiency, coupled with its obligate dependence on specific fungi for germination under natural conditions, further complicates conservation initiatives.^24^

Although mycorrhizal associations have been investigated in congeneric species such as *P. spicerianum,* ^25^ *P. barbigerum,* ^26^ and *P. hirsutissimum,* ^27^ ^,^ ^28^ understanding of the symbiotic fungal communities in wild *P. malipoense* populations remains incomplete. Previous studies have isolated *Tulasnella* strains from cultivated plants,^15^ however, a comprehensive assessment of the functional diversity among these symbiotic fungi—particularly their efficacy in promoting different developmental stages (germination vs. seedling growth)—is still lacking. Given that congeneric orchids often share phylogenetically related mycobionts due to evolutionary conservatism,^29^ it is plausible that functionally effective fungi for *P. malipoense* might also benefit its closely related congeners.

To address the research constraints imposed by the extreme scarcity of *P. malipoense* germplasm resources, an innovative research framework was established. First, symbiotic fungi were isolated from the roots of wild plants. An initial symbiotic germination screening was then conducted using congeneric, readily available species (*P. hirsutissimum* and *P. barbigerum*) as proxies, based on the premise of potential functional overlap in their mycobionts. Finally, the growth‑promoting effects of selected fungal strains were verified directly on *P. malipoense* seedlings. Through this strategy, this study aims to: (1) Assess the functional differentiation among *Tulasnella* strains isolated from *P. malipoense* in facilitating symbiotic germination of two congeneric orchid species, and identify key fungal strains that support complete protocorm-to-seedling development. (2) Quantify the stage-specific effects of the fungi on seed germination and subsequent seedling growth, and determine the correlation between germination promotion capacity and seedling growth enhancement. (3) Elucidate the physiological mechanisms underlying the differential growth promotion by distinct fungal strains, through the analysis of key parameters including nutrient uptake, antioxidant enzyme activities, and photosynthetic characteristics. These findings provide both practical support for *P. malipoense* conservation and insight into stage-specific functional diversity in orchid mycorrhizal fungi.

## Materials and methods

### Experimental materials and sampling

**Figure 1.**
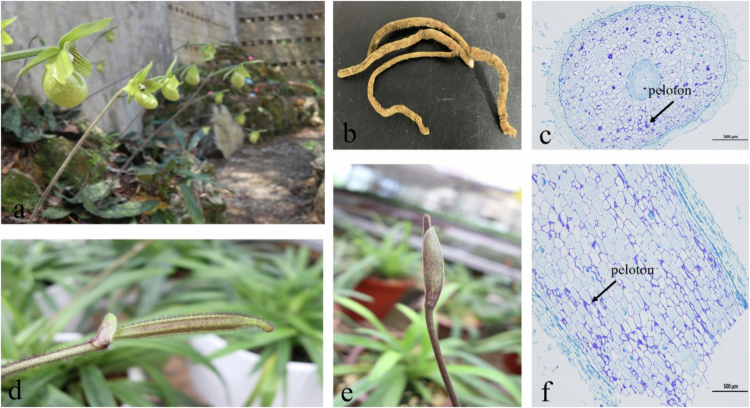
(a) Wild population of *P. malipoense*; (b) Root segment of *P. malipoense*; (c) Transverse section of *P. malipoense* root (arrows indicate pelotons); (d) Capsule of *P. hirsutissimum*; (e) Capsule of *P. barbigerum*; (f) Longitudinal section of *P. malipoense* root.

Root samples were collected from asymptomatic adult *P. malipoense* plants in Wangmo County, Guizhou, China (Figure 1a). Specimens were botanically authenticated as *P. malipoense* by Professor Lianhui Wang. Sampling occurred during peak symbiotic activity (May 2024 to April 2025) across four collection batches. Following IUCN ethical guidelines for threatened flora research and CITES Appendix I restrictions, we minimized impact by harvesting only one root segment (approximately 10 cm length, 1–2 cm diameter; Figure 1b) per individual, with three plants sampled per batch. Fresh segments were immediately sealed in sterile polyethylene bags, transported at 7 °C, and stored at 4 °C in darkness for ≤48 h before processing. Prior to isolation, fresh root cross-sections were microscopically examined to confirm active mycorrhizal colonization, evidenced by the presence of intracellular pelotons within cortical cells (Figure 1c and f). For symbiotic germination assays, seeds of *P. hirsutissimum* and *P. barbigerum* were obtained from greenhouse-cultivated plants at Guizhou Academy of Forestry. To ensure maximal seed viability, mature capsules were harvested immediately prior to dehiscence at 160–180 d (*P. hirsutissimum*) (Figure 1d and 230–250) d (*P. barbigerum*) (Figure 1e) post-pollination.

### Isolation and identification of mycorrhizal endophytic fungi

Fungal endophytes were isolated from root segments using a modified tissue culture protocol. Briefly, fresh roots were rinsed under running deionized water for 2 h to remove adherent soil particles. Surface sterilization was then performed by sequential immersion in 75% (v/v) ethanol for 1 min and 2% (v/v) sodium hypochlorite solution for 5 min, followed by three rinses in sterile distilled water. The efficacy of the surface sterilization procedure was verified by plating 100 μL of the final rinse water onto Potato Dextrose Agar (PDA; containing 200 g/L potato infusion, 20 g/L glucose, and 10 g/L agar) and incubating at 24 °C for 72 h, with no microbial growth observed. Under aseptic conditions, the sterilized roots were sectioned into 1–2 mm segments and placed onto PDA medium. The plates were incubated at 24 °C in darkness. Hyphal tips emerging from the root segments after 5–7 d were excised and transferred to fresh PDA plates for purification.

Purified isolates were point-inoculated onto the center of PDA plates and incubated at 24 °C for 15 d. Colony characteristics, including morphology, pigmentation, and secretion of volatile compounds, were recorded. For microscopic observation, mycelial blocks were inoculated onto fresh PDA plates, and sterile cover slips were inserted at a 45° angle into the medium approximately 3 cm from the inoculation point. After 9–10 d of incubation, when hyphae had colonized approximately 40% of the cover slip surface, the slips were carefully retrieved, mounted on glass slides with a drop of deionized water, and examined under an OLYMPUS BX53 microscope. Hyphal microstructures, particularly the formation of monilioid cells, were documented.^26^

Total genomic DNA was extracted from purified mycelia grown on cellophane-overlaid PDA for 10–15 d using the cetyltrimethylammonium bromide (CTAB) method. The internal transcribed spacer (ITS) region of nuclear ribosomal DNA was amplified by polymerase chain reaction (PCR) using the universal fungal primers ITS1 (5′-TCCGTAGGTGAACCTGCGG-3′) and ITS4 (5′-TCCTCCGCTTATTGATATGC-3′). PCR amplification was performed in a 30 μL reaction volume under conditions described previously.^28^ The amplified products were verified by 1.5% agarose gel electrophoresis and purified. Sanger sequencing of the purified PCR products was conducted by Shanghai Sangon Biotech Co., Ltd. (China).

Taxonomic identification of all rDNA-ITS sequences was conducted using BLAST against the NCBI GenBank database under ≥ 95% sequence similarity criteria. The isolates are deposited at the Biology Research Institute, Guizhou Academy of Forestry. Phylogenetic analysis of rDNA-ITS sequences was conducted in MEGA 12 using the neighbor-joining method with 1000 bootstrap replicates, incorporating 30 fungal isolates comprising 18 NCBI reference strains and 12 representative isolates from this study.

### Cross-species symbiotic seed germination assay

Based on the extremely low pollination rate and fruit set rate of *P. malipoense* seeds, it is difficult to obtain sufficient seeds for germination experiments. Therefore, this study selected seeds of congeneric species—*P. hirsutissimum* and *P. barbigerum* —as research subjects to screen for fungi strains that promote germination, with the objective of screening core fungal resources for the symbiotic germination and seedling cultivation of *P. malipoense*. Based on the morphological diversity, isolation frequency, and phylogenetic analysis of the fungal isolates, this study selected a total of six representative strains for experimentation: four from Clade A (MLP161, MLP116, MLP217, MLP027) and two from Clade B (MLP221, MLP232). These strains were used to quantitatively analyze their effects on the seed germination rates of two closely related *Paphiopedilum* species, with the aim of preliminarily evaluating their germination-promoting function. The mycelium of each fungal isolate was inoculated onto 30 mL of PDA medium within a 200 mL tissue culture bottle. This procedure was repeated in triplicate per treatment, with uninoculated PDA serving as the control (CK). All bottles were incubated in darkness at 25 °C ± 2 °C. After two weeks, the fungal-colonized media were used for symbiotic germination assays.

For seed preparation, one mature and well-developed fruit capsule was surface-sterilized sequentially 75% (v/v) ethanol for 3 min and 2% (v/v) sodium hypochlorite solution for 15 min, followed by three rinses in sterile distilled water. The capsule was then aseptically dissected using sterile scalpel and forceps. Seeds were released by gentle shaking and uniformly sown onto the pre-cultured fungal-PDA medium at a density of approximately 300 seeds per bottle. Germination cultures were maintained at 25 °C ± 2 °C under a 12 h photoperiod (1200 lux light intensity/12 h dark cycle).

Developmental stages were classified according to Rasmussen^30^ into five distinct phases: (1) Imbibition (characterized by testa softening and swelling); (2) Testa rupture (marked by embryo extrusion); (3) Protocorm formation (development of spherical green structures); (4) Organ differentiation (initiation of primordia); and (5) Seedling establishment (formation of functional roots and leaves). Following 15 d post-inoculation (dpi) culture period, germination progress and developmental stages were assessed in tissue culture bottles at regular intervals (e.g., weekly and monthly) throughout dark incubation. Data from three biological replicate bottles were averaged for subsequent quantitative analyses. Germination rate was calculated using the formula: (Number of germinated seeds/Total inoculated seeds) × 100%.

### Establishment of seedling-fungus co-culture system

Building on the germination screening results, this subsequent phase of the study focused on investigating the differential effects of various fungal strains on seedling establishment and physiological functions. To assess the developmental stage specificity of the fungal functions, five core strains exhibiting distinct phenotypic outcomes from the germination assay were selected for in-depth comparison. These included high-efficiency germination-promoting strains (MLP027 and MLP232), strains that only initiated germination but failed to support protocorm development (MLP161 and MLP217), and a strain that supported protocorm formation but induced seedling leaf yellowing (MLP221). Furthermore, to broaden the functional assessment and explore potential unique physiological effects, two additional strains (MLP281 and MLP246) were included based on their distinctive morphological characteristics in pure culture (Figure 2). A non-inoculated blank control group (CK) was also set up in parallel to systematically analyze the symbiotic functions of different strains at the seedling stage.

Accordingly, mycelial plugs (5 mm in diameter) were obtained from each strain using a cork borer and individually inoculated into tissue culture bottles containing PDA medium for pre-culture. After the mycelia had fully colonized the medium, uniformly grown one-year-old *in vitro* seedlings of *P. malipoense* were transplanted into the fungus-inoculated culture bottles. Non-inoculated treatments served as the control group. The experiment followed a completely randomized block design with ten replicate culture bottles per treatment, each containing two seedlings, totaling 20 seedlings per treatment. All culture bottles were maintained in a growth chamber under controlled conditions (25 °C/20 °C d/night temperature, 12 h photoperiod, 150 μmol·m⁻²·s⁻¹ light intensity) for a three-month co-culture period.

### Measurement of growth and physiological parameters

Following a three-month symbiotic cultivation period, five mycorrhizal seedlings with uniform growth status were randomly selected from each treatment group for systematic measurement of growth parameters. Growth indices included seedling height, maximum leaf length and width, taproot length (measured using a ruler), and taproot diameter (measured using a Vernier caliper). The entire fresh weight of each seedling was subsequently determined using an analytical balance.

For physiological indices, fresh leaf tissue samples were used. Notably, seedlings in the CK cultured on PDA medium did not develop and gradually browned, likely due to the lack of symbiotic fungal support and potential nutrient stress. Consequently, these seedlings failed to yield sufficient leaf tissue for physiological analysis. Therefore, the comparative analysis of physiological characteristics in this study focused on evaluating the differential effects among the various fungal treatments. After collection, samples were immediately frozen in liquid nitrogen and stored at –80 °C in an ultra-low temperature freezer. Subsequent procedures were carried out according to the protocols provided with the respective assay kits from Nanjing Mofan Biotechnology Co., Ltd.^31^ Antioxidant enzyme activities were determined as follows: Peroxidase (POD) activity was measured by monitoring the change in absorbance at 470 nm over 5 min using guaiacol as the substrate. Catalase (CAT) activity was determined using the ammonium molybdate method, based on the principle that the enzyme decomposes H₂O₂, and the residual H₂O₂ reacts with ammonium molybdate to form a light-yellow complex, with the absorbance measured at 405 nm. Superoxide dismutase (SOD) activity was assessed via the WST-8 method, which evaluates the enzyme's inhibitory effect on the reduction of WST-8 by superoxide anions to form a formazan dye, with the absorbance measured at 450 nm. All enzyme activities are expressed on a fresh weight basis.

Chlorophyll content was determined using the acetone extraction method. Fresh leaf samples were ground under low-light conditions and extracted with acetone until the tissue residue was completely bleached. The absorbance of the extract was then measured at 645 nm and 663 nm using a spectrophotometer, and the total chlorophyll content was calculated using empirical formulas. Results are expressed as milligrams per gram fresh weight. Nitrate reductase (NR) activity was measured by an *in vitro* assay. The method is based on the enzyme's catalysis of nitrate reduction to nitrite. The nitrite produced under acidic conditions forms a red azo compound, the absorbance of which is measured at 540 nm. Enzyme activity is defined as the amount that catalyzes the production of 1 μmol of NO₂^−^ per hour per gram fresh weight. The determination of total nitrogen and total phosphorus contents was performed with reference to the Chinese Agricultural Industry Standard NY/T 2017–2011.^32^ Plant samples were dried at 65 °C to constant weight and ground into a powder. Total nitrogen content was determined using the Kjeldahl method, and total phosphorus content was measured by the molybdenum-antimony anti-spectrophotometric method.

Indole-3-acetic acid (IAA) content in plant tissues was determined using high-performance liquid chromatography with fluorescence detection. Approximately 0.2 g of fresh tissue sample was accurately weighed and homogenized with 1 mL of pre-cooled 80% methanol aqueous solution containing 1% acetic acid. The homogenization was performed at 4 °C under light-protected conditions. The homogenate was then stored overnight at 4 °C in the dark for extraction. After centrifugation, the supernatant was collected and subjected to sequential purification steps: triple extraction with 0.5 mL petroleum ether for decolorization (discarding the upper ether phase), pH adjustment to 2.8 by adding 0.06 mL saturated citric acid, followed by triple extraction with 0.5 mL ethyl acetate with combination of the organic phases. The combined organic phases were evaporated to dryness under a nitrogen stream, reconstituted in 0.5 mL methanol with vortex mixing, and filtered through a 0.22 μm microporous membrane before injection. Chromatographic separation was carried out on a C18 reverse-phase column maintained at 30 °C, using a mobile phase of water-methanol-acetic acid mixture at a flow rate of 0.8 mL/min. Fluorescence detection was set at excitation and emission wavelengths of 275 nm and 345 nm, respectively. IAA content was quantified by the external standard method and expressed as nanograms per gram fresh weight. Soluble protein content was determined using a bicinchoninic acid (BCA) protein assay kit.

### Statistical analysis

All experimental data are presented as the mean ± standard deviation from at least three biological replicates. Statistical analyses were performed using one-way analysis of variance (ANOVA) in SPSS Statistics 27.0, followed by Tukey's honestly significant difference (HSD) post hoc test for multiple comparisons. Differences were considered statistically significant at *p* < 0.05. All figures were generated using GraphPad Prism version 10.1.2.

## Results and analysis

**Figure 2.**
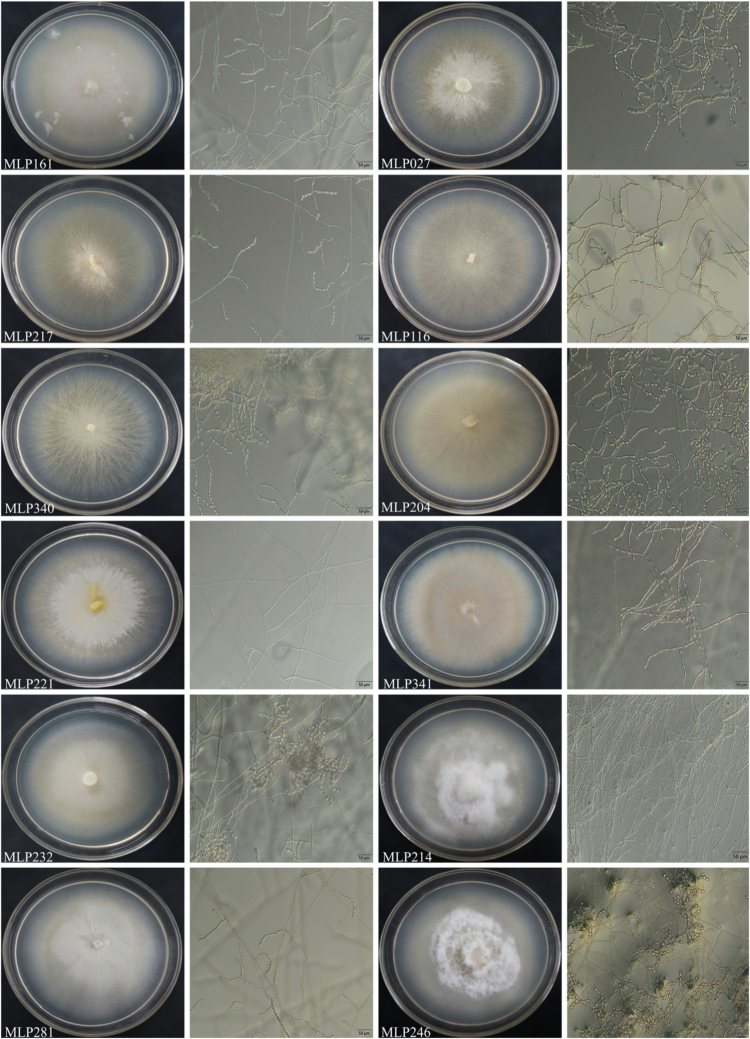
Representative colony morphology of 12 culturable fungal endophytes isolated from wild *P. malipoense* roots following 15 d of incubation on PDA at 24 °C, showing isolates MLP161, MLP027, MLP217, MLP116, MLP340, MLP204, MLP221, MLP341, MLP232, MLP214, MLP281, and MLP246 (MLP = *P. malipoense*).

### Morphological characteristics and molecular identification of culturable endophytic fungi from *P. malipoens*e roots

Culture-dependent isolation from root systems of *P. malipoense* yielded 12 culturable fungal strains exhibiting morphological characteristics presumptively identified as *Tulasnella*, including monilioid cells (representative strains MLP027, MLP246). Following 15 d of incubation on PDA at 24 °C, isolates could be broadly categorized into two distinct morphotypes based on integrated assessment of colonial architecture and micromorphological features (Figure 2). Group 1 strains (MLP161, MLP027, MLP217, MLP116, MLP340, MLP204) developed cream-white colonies exhibiting floccose to dendritic mycelial growth with entire margins, all producing oblong monilioid cells. Notably, MLP027 formed abundant elliptical monilioid cells, while MLP217 developed only rudimentary structures. During late-stage growth, white waxy protuberances emerged at the centers of MLP027, MLP217, and MLP340 colonies. Additionally, Group 2 strains (MLP221, MLP341, MLP232, MLP214, MLP281, MLP246) formed adherent, compact cream-white colonies with regular margins and prominent concentric zonation. All Group 2 strains except MLP214 and MLP221 differentiated monilioid cells to varying degrees, MLP232 and MLP246 produced abundant spherical monilioid cells, whereas MLP214 and MLP221 formed exclusively chain-like structures. Both MLP214 and MLP246 additionally developed cottony aerial mycelia in central regions during late growth stages. Morphological delineation of *Tulasnella* species remains challenging due to limited diagnostic characters among taxa and difficulties in inducing sexual spores.^33^

**Table 1.** Taxonomic affiliation of fungal endophytes from *P. malipoense* roots based on rDNA-ITS sequence similarity against the GenBank database.

Fungal isolate	Isolation frequency (%)	rDNA-ITS sequence alignment result	Coverage (%)	Identity (%)	Closest match in GenBank
MLP161	7.60	*Tulasnella* sp. isolate parm31	100	97.05	OQ678371.1
MLP116	12.28	*Tulasnella* sp. isolate pwen31	99	99.06	OQ678370.1
MLP217	7.90	*Tulasnella* sp. isolate pjack49	98	96.23	OQ678361.1
MLP027	15.50	*Tulasnella* sp. isolate pjack49	98	96.35	OQ678361.1
MLP214	5.56	*Tulasnella* sp. isolate TCA3	100	99.67	OP740390.1
MLP340	7.02	*Tulasnella* sp. Isolate Dfrh-2	100	100	OR879206.1
MLP341	6.45	*Tulasnella* sp. isolate pjack49	97	96.08	OQ678361.1
MLP204	5.26	*Tulasnella* sp. isolate pjack49	100	95.92	OQ678361.1
MLP246	4.97	*Tulasnella* sp. isolate TCA2	100	99.51	OP740384.1
MLP232	10.82	*Tulasnella* sp. isolate TCA3	100	99.67	OP740390.1
MLP221	10.23	*Tulasnella* sp. isolate TCA4	100	99.35	OP740400.1
MLP281	6.14	*Tulasnella* sp. isolate pemer202	96	99.32	OQ678398.1

BLAST analysis confirmed that all 12 fungal isolates shared highest sequence similarity with *Tulasnella* species, demonstrating average query coverage of 99% and sequence identity of 98.19% (Table 1). Significantly, several isolates showed closest homology to *Tulasnella* reference sequences OQ678361.1 and OP740390.1, which were originally obtained from greenhouse-cultivated *P. malipoense* roots.^15^ This molecular congruence supports their ecological function as core symbionts of *P. malipoense*.

**Figure 3.**
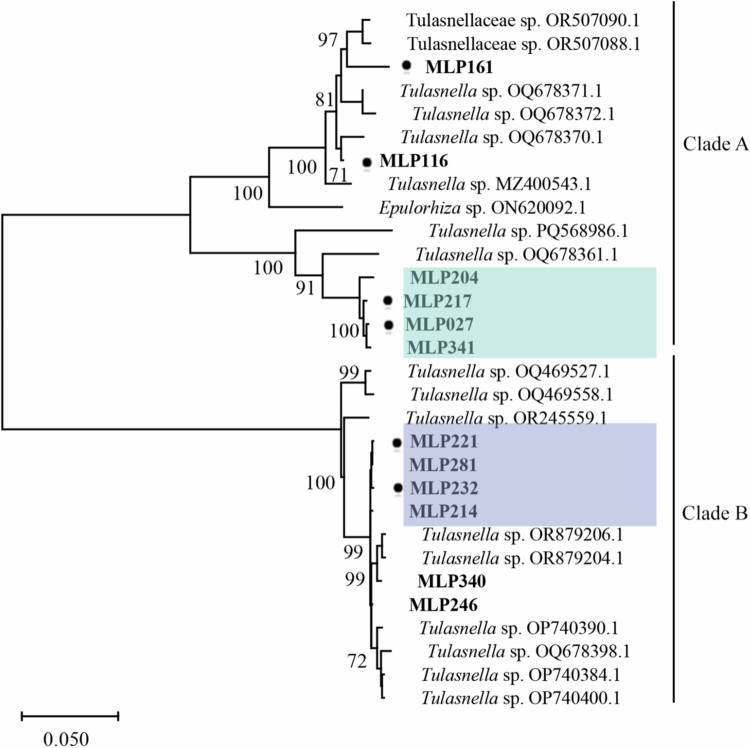
Phylogenetic relationships of root endophytes in *P. malipoense* inferred from rDNA-ITS sequence analysis. Bootstrap support values > 70% are shown at nodes. Color blocks highlight the two potential subclade. Strains marked with black dots were employed for symbiotic germination assays.

The resulting phylogeny (Figure 3) strongly supported BLAST identifications, confirming exclusive clustering of all root-associated fungi within *Tulasnella* species. The isolates formed two well-supported monophyletic clades: Clade A (6 isolates) and Clade B (6 isolates). Within Clade A, a distinct subclade containing MLP204, MLP217, MLP027, and MLP341 exhibited substantial divergence with 100% bootstrap support. Similarly, a divergent Clade B subclade comprising MLP221, MLP281, MLP232, and MLP214 received strong nodal support (99% bootstrap), may represent novel taxa, this requires confirmation through multi-locus analysis.

### Promotive effect of specific strains on seed germination of *Paphiopedilum* species

**Table 2.** Symbiotic germination efficacy of different fungal isolates on *P. barbigerum* and *P. hirsutissimum* after 90 d of co-culture.

Strains	Germination and growth stage				
	Stage 1	Stage 2	Stage 3	Stage 4	Stage 5
MLP161	0.4349 ± 0.0062b	0.0000 ± 0.0000b	0.0000 ± 0.0000b	0.0000 ± 0.0000b	0.0000 ± 0.0000b
MLP116	0.7496 ± 0.0214a	0.6378 ± 0.0223a	0.6223 ± 0.0224a	0.6064 ± 0.0292b	0.5427 ± 0.0062a
MLP221	0.7314 ± 0.0287a	0.6463 ± 0.0609a	0.6385 ± 0.0601a	0.5849 ± 0.0548a	0.5359 ± 0.0415a
CKXY	0.1427 ± 0.0071c	0.0000 ± 0.0000b	0.0000 ± 0.0000b	0.0000 ± 0.0000b	0.0000 ± 0.0000b
MLP027	0.8259 ± 0.0154a	0.7051 ± 0.0062a	0.6376 ± 0.0180a	0.5737 ± 0.0064a	0.5488 ± 0.0101a
MLP232	0.8383 ± 0.0224a	0.7356 ± 0.0241a	0.6937 ± 0.0186a	0.6359 ± 0.0190a	0.6201 ± 0.0147a
MLP217	0.5058 ± 0.0353b	0.0000 ± 0.0000b	0.0000 ± 0.0000b	0.0000 ± 0.0000b	0.0000 ± 0.0000b
CKDY	0.2322 ± 0.0266c	0.1136 ± 0.0057b	0.0000 ± 0.0000b	0.0000 ± 0.0000b	0.0000 ± 0.0000b

Note: data represent the proportion of seeds at each developmental stage (mean ± SD, *n* = 3). Stages: 1, imbibition; 2, testa rupture; 3, protocorm; 4, leaf primordia; 5, seedling. The experiment comprised two independent host–fungus systems: *P. barbigerum* (MLP161, MLP116, MLP221, CKXY) and *P. hirsutissimum* (MLP027, MLP232, MLP217, CKDY). Within each system and stage, different lowercase letters denote significant differences (*p < *0.05). Cross-system or cross-stage comparisons are not statistically valid.

Inoculation with mycorrhizal fungi significantly accelerated seed germination progression in both *P. barbigerum* and *P. hirsutissimum* relative to uninoculated controls (*p* < 0.05, Table 2). Control groups exhibited developmental constraints: *P. barbigerum* seeds achieved only 14.27% imbibition with testa rupture but failed to form protocorms, while *P. hirsutissimum* showed 23.22% imbibition and 11.36% protocorm formation without subsequent advancement. Strain-specific effects were observed across developmental stages, demonstrating strict ontogenetic stage dependency.

**Figure 4.**
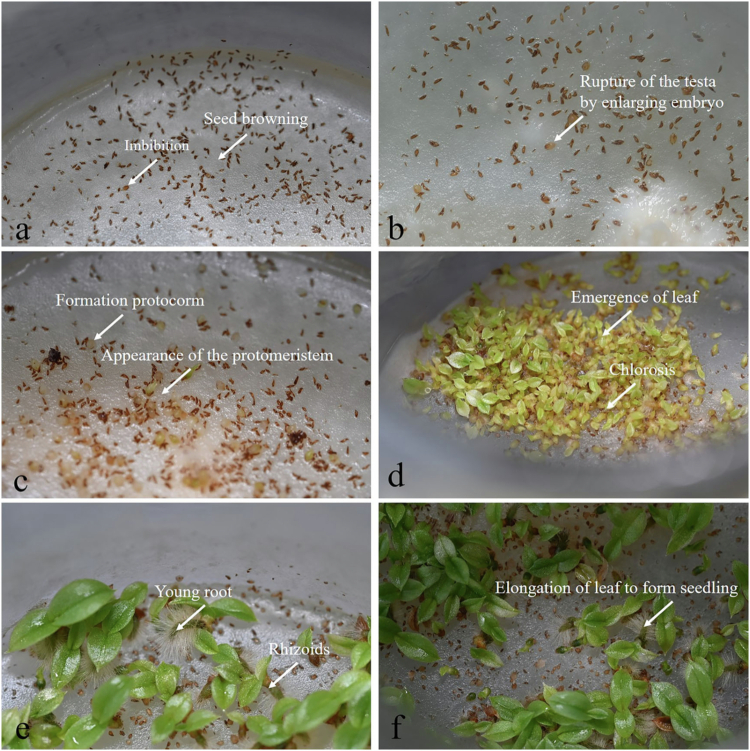
Symbiotic germination of *P. barbigerum* seeds inoculated with PDA-cultured *Tulasnella* strains MLP161, MLP116, and MLP221. Key developmental stages captured include: (a) seed imbibition with visible testa swelling, (b) testa rupture accompanied by embryo extrusion, (c) formation of spherical green protocorms, (d) emergence of leaf primordia, and (e) developed seedling exhibiting ciliate rhizoids and (f) photosynthetically active leaves.

For *P. barbigerum*, isolate MLP116 demonstrated higher symbiotic efficacy. Universal testa rupture occurred at 30 dpi (Figure 4b), followed by protocorm development in 63.78% of seeds by 45 dpi (Figure 4c). Paired leaf primordia emerged at 60 dpi (Figure 4e), culminating in 54.27% of seeds establishing autotrophic seedlings with functional root systems by 90 dpi (Figure 4f). Microscopic observation confirmed the formation of intracellular pelotons in root cortical cells. By contrast, the seeds inoculated with MLP161 showed discoloration after imbibition (Figure 4a) and failed to germinate. Although MLP221 initiated protocorm formation in 64.63% of seeds at 45 dpi, subsequent leaf chlorosis and developmental arrest at 90 dpi (Figure 4d) prevented seedling establishment.

In *P. hirsutissimum*, isolates MLP027 and MLP232 facilitated complete ontogenetic progression. Testa rupture was observed at 30 dpi, with protocorm formation rates reaching 70.51% and 73.56% respectively at 45 dpi—significantly surpassing MLP217 (0%). Primary leaf primordia differentiated at 60 dpi, advancing to secondary leaf primordia and rhizoid development by 90 dpi. Final seedling establishment rates reached 54.88% (MLP027) and 62.01% (MLP232) (Table 2), confirming their status as optimal symbionts. MLP217-supported specimens arrested development immediately post-testa rupture without protocorm initiation.

#### Fungal inoculation promotes seedling growth in *P. malipoense*

**Figure 5.**
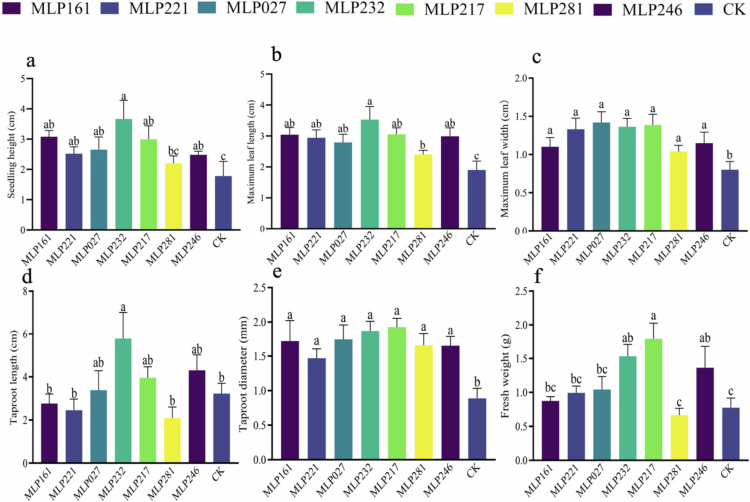
Promotional effects of mycorrhizal fungi on the growth of *P. malipoense* seedlings at 90 d post-inoculation. The figure illustrates the impact of co-cultivation with PDA medium on seedling development. Panels display mean values of (a) seedling height, (b) maximum leaf length, (c) maximum leaf width, (d) taproot length, (e) taproot diameter, and (f) fresh weight. Means labeled with different letters indicate statistically significant differences (*p* < 0.05).

Following a three-month symbiotic cultivation period, we systematically evaluated the growth and physiological parameters of *P. malipoense* seedlings. Compared to the control group, most fungal inoculation treatments exhibited varying degrees of growth promotion, with significant differences observed among the different fungal strains (*p* < 0.05, Figure 5). Among them, strains MLP232, MLP217, MLP027, and MLP246 demonstrated the most pronounced growth-promoting effects. In terms of phenotypic growth, the MLP232 and MLP217 treatment groups showed outstanding performance. Their seedling height (Figure 5a), maximum leaf length (Figure 5b), and taproot length (Figure 5d) were significantly greater than those of both the control group and the MLP281 treatment group. Regarding maximum leaf width (Figure 5c) and taproot diameter (Figure 5e), although no significant differences were observed among the different treatment groups, all values were significantly higher than those of the control group (*p* < 0.05). Biomass analysis revealed that the MLP217, MLP232, and MLP246 treatment groups resulted in the most significant increases in seedling fresh weight, which were 131%, 98%, and 76.2% higher than the control group, respectively (*p* < 0.05, Figure 5f). Notably, the fresh weight of seedlings in the MLP281 treatment group was numerically lower than, but not statistically different from, that of the control group (Figure 5f). Further observation indicated that during the symbiotic process, the mycelium of this strain excessively enveloped the seedlings, potentially hindering their normal growth and development.

#### Effects of different symbiotic fungal strains on physiological characteristics of *P. malipoense* seedlings

**Figure 6.**
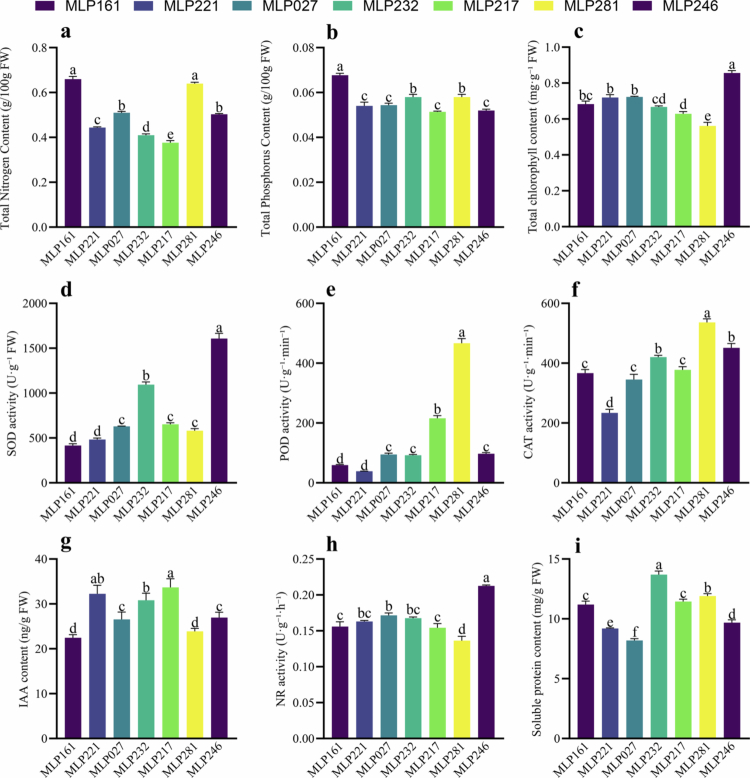
Effects of different symbiotic fungal strains on physiological characteristics of *P. malipoense* seedlings at 90 d post-inoculation. Panels display mean values of (a) total nitrogen content, (b) total phosphorus content, (c) total chlorophyll content, (d) superoxide dismutase (SOD) activity, (e) peroxidase (POD) activity, (f) catalase (CAT) activity, (g) nitrate reductase (NR) activity, (h) soluble protein content, and (i) indole-3-acetic acid (IAA) content. Means labeled with different letters indicate statistically significant differences (*p* < 0.05).

Following establishment of symbiotic relationships with different fungal strains, significant alterations were observed in nutrient acquisition, photosynthetic characteristics, antioxidant enzyme systems, and nitrogen metabolism-related indicators in *P. malipoense* seedlings (*p* < 0.05; Figure 6). Regarding nutrient element accumulation, distinct strain-specific effects were evident among the different fungal isolates. Total nitrogen content reached its highest levels in the MLP161 and MLP281 treatments, significantly exceeding other strains, followed by MLP027 and MLP246, while MLP217 showed the lowest total nitrogen content (Figure 6a). Total phosphorus content was significantly higher in MLP161, MLP232, and MLP281 treatments compared to other groups (Figure 6b). Fungal strains also differentially influenced leaf photosynthetic pigment content, with MLP246-treated plants exhibiting the highest total chlorophyll content, followed sequentially by MLP027, MLP221, and MLP161 treatments, while MLP281 treatment resulted in significantly lower chlorophyll content than other strains (Figure 6c).

The symbiotic fungi similarly regulated the oxidative homeostasis system within the host plants (Figure 6d–f). MLP246 treatment significantly enhanced SOD and CAT activities, though its POD activity remained at relatively low levels. MLP232 treatment concurrently enhanced both SOD and CAT activities. Notably, although MLP281 treatment was associated with lower SOD activity, it induced the highest POD and CAT activities, suggesting that different fungal strains may influence the host's reactive oxygen species metabolism through distinct regulatory mechanisms.

In terms of hormone and nitrogen metabolism, the fungal strains also exhibited different regulatory patterns. IAA content was highest in MLP217 and MLP221 treatments, followed by MLP232 and MLP246 treatments, while MLP161 and MLP281 treatments showed the lowest values (Figure 6g). NR activity was highest in MLP246 treatment but significantly suppressed in MLP281 treatment (Figure 6h). Additionally, soluble protein content varied significantly among treatments, displaying the following order: MLP232 > MLP281 > MLP217 > MLP161 > MLP246 > MLP221 > MLP027 (Figure 6i).

## Discussion

### Specificity of the cultivable mycobiota in wild *P. malipoense* roots

Culture-dependent isolation from the roots of wild *P. malipoense* yielded a mycobiota exclusively composed of fungi from the genus *Tulasnella*. All 12 obtained isolates were morphologically preliminarily identified as *Tulasnella* based on features such as colony morphology and the presence of monilioid cells. Molecular identification based on rDNA-ITS sequences confirmed this taxonomic assignment, with all isolates showing high sequence similarity (95.92%–100%) to known *Tulasnella* species in GenBank (Table 1). The inherent limitations of rDNA-ITS markers for species discrimination, coupled with sparse genetic database coverage of orchid mycorrhizal fungi, precluded species-level identification. Phylogenetic analysis further resolved these isolates into two distinct, well-supported clades (Clade A and Clade B), indicating phylogenetic diversity within this specific fungal guild, future investigations should implement multi-locus phylogenetic approaches to delineate the taxonomic status of these subclades.^13^

The congruence between our isolates from wild plants and previously reported *Tulasnella* sequences from cultivated *P. malipoense* ^15^ reinforces the conclusion that *Tulasnella* represents a core, and under our cultivation conditions, the predominant cultivable mycobiont associated with this orchid species. This finding of low cultivable diversity aligns with patterns of high mycorrhizal specificity observed in other *Paphiopedilum* species.^14^ ^,^ ^15^ This specificity is likely associated with developmental stages, as the fungal community composition shifts from mycoheterotrophic in juveniles to autotrophic in adults during plant development.^34^ ^,^ ^35^ Besides, we hypothesize that habitat specialization likely drives obligate symbiotic specificity between *P. malipoense* and particular *Tulasnella* fungi.^36^ The species' harsh karst habitat features high alkalinity and phosphorus deficiency,^37^ creating abiotic filters that select for symbiotic fungi resilient to geochemical constraints. Empirical studies confirm that specific *Tulasnella* strains maintain efficient phosphatase activity for phosphorus acquisition under alkaline conditions.^38^ Additionally, substantial evidence further indicates that species-specific signaling molecules mediate *Tulasnella*-orchid symbiosis.^39^ ^,^ ^40^ These compounds facilitate precise partner recognition through mechanisms including directed hyphal growth and host defense suppression,^41^ promoting symbiotic establishment. The synergy between abiotic filtering and biochemical signaling likely underpins ecological dominance of specific *Tulasnella* strains within *P. malipoense*-associated fungal communities. Resolving the molecular structures of these signaling compounds and their spatiotemporal dynamics at symbiotic interfaces will require advanced techniques such as *in situ* metabolomics.

### Strain-specific and stage-dependent efficacy in symbiotic germination

Cross-species germination assays revealed substantial strain-specificity among *Tulasnella* isolates in promoting seed germination and seedling development.^16^ ^,^ ^18^ This functional divergence corresponds to shifting host nutritional demands: protocorms rely on fungal carbon, while mature plants depend on mineral nutrients.^35^ Only MLP116, MLP027, and MLP232 supported complete protocorm-to-seedling development (establishment rates: 54.27%, 54.88%, and 62.01%, respectively), likely through successful colonization and nutrient provision.^8^ In contrast, MLP161 and MLP217 only supported imbibition, while MLP221 initiated protocorm formation but failed to sustain subsequent development, indicating stage-specific functional deficiencies.^16^

At the molecular level, compatible fungal strains induce host metabolic reprogramming prior to physical contact through lipid-like signaling molecules.^39^ ^,^ ^41^ ^,^ ^42^ Subsequent modulation of effector molecules facilitates host cell wall remodeling, expanding symbiotic interfaces for nutrient translocation.^43^ Multiple phytohormones, including IAA, JA, ABA, and SLs, coordinately regulate symbiotic development from germination to seedling establishment.^44^ ^,^ ^45^ In this study, we observed significantly elevated IAA levels in leaves of *P. malipoense* seedlings colonized by fungal strains such as MLP232 and MLP217, indicating an association between endogenous IAA and fungal activity. However, despite showing the highest IAA content, the MLP217 treatment did not exhibit germination‑promoting activity, revealing the complexity of symbiotic regulation. These results suggest that symbiotic outcomes are not determined solely by the abundance of a single hormone such as IAA, but rather depend on a specific hormonal context integrated with other fungal‑derived contributions, such as carbohydrate supply. Future studies will employ transcriptomic and metabolomic approaches to further elucidate the molecular mechanisms through which strains like MLP232 exert their effects. In summary, our cross-species screening identified core candidate strains (MLP116, MLP027, MLP232) with high symbiotic potential. While direct effects on *P. malipoense* germination require validation, their demonstrated ability to complete life cycles in congeneric species and enhance seedling growth supports their conservation application.

### Functional decoupling and physiological trade-offs in seedling symbiosis

Symbiotic cultivation of *P. malipoense* seedlings showed that most fungal strains promoted growth, yet a clear functional dissociation existed between germination promotion and seedling growth enhancement.^46^ Specifically, MLP217, while ineffective during germination, increased seedling fresh weight by 131% (Figure 5f) and induced high IAA levels. In contrast, the strong germination-promoter MLP232 also substantially enhanced growth (98% fresh weight increase), with elevated IAA and soluble protein (Figure 6g, i), indicating multi-stage symbiotic capacity consistent with compatible *Tulasnella* strains.^47^

Physiological profiling revealed distinct functional specialization among *Tulasnella* strains in symbiosis with *P. malipoense*. Growth-promoting strains MLP217 and MLP232 drove substantial biomass accumulation yet showed lower tissue nitrogen and chlorophyll concentrations (Figure 6a and c), reflecting the “dilution effect” of rapid growth.^48^ In contrast, MLP281 suppressed seedling development but elevated antioxidant enzymes (POD and CAT, Figure 6e and f), while enhancing nitrogen and soluble protein content (Figure 6a and i), suggesting resource reallocation toward defense mechanisms.^49^ MLP161 specifically improved plant nitrogen and phosphorus acquisition (Figure 6a and b) without enhancing growth, indicating specialized nutrient facilitation.

These results demonstrate a functional trade-off in the *Tulasnella-Paphiopedilum* symbiosis: strains excelling in growth promotion tend to be less effective in enhancing nutrient concentration or stress resistance. This physiological evidence highlights the strategic diversity in orchid-fungal symbiotic relationships.

## Conclusion and prospects

This study elucidates that the root-associated mycobiota of wild *P. malipoense* is dominated by functionally diverse *Tulasnella* strains. This study not only identified specific strains with high efficacy in promoting cross-species germination (MLP116, MLP027, MLP232) and seedling growth (MLP217, MLP232, MLP246), but also critically revealed a developmental stage-dependent functional decoupling between these traits. This functional specialization underscores the limitation of single-strain inoculation. Consequently, future conservation efforts should prioritize: (1) Validating the efficacy of these core strains on *P. malipoense* seeds per se; (2) Employing multi-omics approaches to decipher the mechanistic basis of growth promotion by strains like MLP217, particularly their role in nutrient mobilization and stress tolerance; and (3) designing and testing synthetic microbial consortia comprising functionally complementary strains to orchestrate the entire early developmental process of this endangered orchid.

## Data Availability

In response to the reviewer's suggestion, the ITS sequences of the 12 *Tulasnella* fungal strains involved in this study have been submitted to the NCBI GenBank database, with accession numbers pending (Submission ID: SUB15806500). The data will be made publicly available immediately upon formal acceptance of the article, and the corresponding accession numbers will be provided in the final proof. To support the peer review process, the complete sequence dataset is available from the corresponding author under a confidentiality agreement. Elite fungal strains: Deposited at the Biology Research Institute, Guizhou Academy of Forestry, China.

## Abbreviations

MLP: *Paphiopedilum malipoense*
PDA: potato dextrose agar
CTAB: cetyltrimethylammonium bromide
rDNA-ITS: internal transcribed spacer region of nuclear ribosomal DNA
PCR: polymerase chain reaction
dpi: days post-inoculation
IAA: indole-3-acetic acid
SOD: superoxide dismutase
POD: peroxidase
CAT: catalase
NR: nitrate reductase
